# When emergency care starts at the door: Removing financial barriers to timely treatment in fragile health systems

**DOI:** 10.1016/j.afjem.2026.101025

**Published:** 2026-10-23

**Authors:** Omar Ismail Mohamed, Abdirahman Mohamed Adan, Salma Zubair Mohamud, Haadi Qaasim Barre, Sadio Abdulahi Osman

**Affiliations:** a Faculty of Health Sciences and Tropical Medicine, Somali National University, Mogadishu, Somalia; b Center for Health Research and Innovation, Somali National University, Mogadishu, Somalia

**Correspondence**

A patient can reach an emergency facility and still be denied timely care if payment is required before triage, assessment, stabilization, diagnostics, medicines, referral, or admission. In emergency medicine, this is not only a financing problem; it is a patient-safety problem because time-critical illness deteriorates while administrative and payment checkpoints are negotiated [1,2]

This distinction matters in fragile health systems, where out-of-pocket spending, weak prehospital care, limited emergency capacity, and medicine and diagnostic shortages already compress the margin for safe delay [1,3] . Payment requirements can delay care at several points: families may postpone seeking help while raising funds; registration or deposits may slow entry into emergency units; financial clearance may postpone oxygen, fluids, tests, or essential medicines; and inability to pay may interrupt referral, admission, or definitive treatment [1] . Qualitative evidence from Tanzania describes the same pathway: upfront payments delayed diagnostics, medications, and treatment, sometimes for hours or longer, with reported progression to disability or death [2]

Somalia illustrates why this issue is especially important in Africa’s fragile and conflict-affected settings. A nationwide hospital assessment found that more than 80% of hospitals were not ready to provide emergency and critical care, only 17.6% reported formal triage systems, and user fees were the most frequently cited barrier to emergency readiness [3] The same study found particularly limited availability of oxygen, blood transfusion capacity, and 24-hour staff presence, meaning even short payment-related delays can become clinically consequential [3] Recent regional evidence also shows that financial barriers are especially acute in Somalia, where 64.8% of women reported money for treatment as a major barrier, reflecting the broader interaction of poverty, underfunding, and reliance on out-of-pocket payment [4]

The policy response should therefore start at the emergency door. First, facilities and ministries should adopt triage-first and stabilize-first rules that prohibit payment requirements from delaying emergency assessment and life-saving stabilization [1] Second, these policies need a financing mechanism: ring-fenced stabilization funds, emergency waivers, or reimbursement for uncompensated emergency care can reduce dependence on bedside deposits in underfinanced hospitals [1] Third, hospitals should separate billing from clinical triage and protect essential diagnostics, medicines, oxygen, and referral from immediate payment barriers [1] This approach is feasible: in Cameroon, providing essential trauma supplies without upfront payment was associated with shorter time to emergency disposition and substantially less patient-reported financial interference with care [5]

This can be described as financial triage: when ability to pay effectively influences whether, or how quickly, emergency care proceeds. Emergency systems cannot be considered timely, equitable, or high quality if financial clearance becomes part of triage. Financial protection is not separate from emergency-care quality; when payment barriers delay emergency treatment, financing becomes part of the clinical pathway [6]

Financial barriers can delay or prevent timely access to emergency assessment, stabilization, diagnostics, medicines, and referral, particularly in fragile and conflict-affected health systems. This correspondence reframes upfront payment and other financial barriers as emergency-care and patient-safety issues, rather than solely health-financing problems. Using Somalia as an illustrative context, it highlights practical approaches to protect timely emergency care from ability-to-pay barriers and offers a broader policy message for fragile African health systems.

## Declaration of competing interest

The authors declare that they have no known competing financial interests or personal relationships that could have appeared to influence the work reported in this manuscript.
